# Moving hens into cages affects cognitive performance, extinction learning and motivation for rewards

**DOI:** 10.1017/awf.2026.10084

**Published:** 2026-04-14

**Authors:** Javiera Calderón-Amor, Benjamin Lecorps, Rodrigo Ávila, Tamara Tadich

**Affiliations:** 1 Escuela de Graduados, Universidad Austral de Chile, Chile; 2 Facultad de Agronomía y Sistemas Naturales, Pontificia Universidad Católica de Chile, Chile; 3 Bristol Veterinary School, United Kingdom; 4 Universidad Austral de Chile Facultad de Ciencias Veterinarias, Chile; 5 Ciencia Animal, Universidad Austral de Chile, Chile

**Keywords:** animal welfare, behavioural plasticity, cognitive performance, *Gallus gallus domesticus*, housing system, motivation

## Abstract

Understanding chicken cognition is essential for improving welfare in production systems, as it reveals how animals perceive and respond to their environment. Barren housing can compromise welfare, including negative affective states and cognitive deficits, but previous research mostly focused on effects of barren environments on young animals. Here, we investigated whether hens moved to battery cages once adults show lower cognitive performance than those kept cage-free. Because stable inter-individual differences (personality traits) can modulate how animals respond to environmental changes, we also explored whether personality modulates this effect. Sixty hens were reared in enriched aviary pens; at 18 weeks, half were transferred to battery cages (456 cm² per hen) and the rest remained cage-free (5,333 cm² per hen) for 64 days before testing. Personality was assessed through four standardised tests, and spatial memory was evaluated with a modified hole-board task. Working memory (WM), general working memory (GWM), and reference memory (RM) were calculated from visit ratios. Behaviours after birds consumed all baited rewards were recorded to assess responses to reward omission (e.g. extinction learning). Battery hens outperformed cage-free hens in all memory metrics and were more active during the post-reward period, showing more empty-cup visits. While the enhanced memory performance of battery hens is likely driven by increased reward motivation and greater engagement with the task, our results also suggest these birds showed a deficit in extinction learning. Personality also influenced performance: more fearful hens had lower WM and GWM and were slower to find baited cups. Housing and personality jointly shaped cognition in laying hens, highlighting that enhanced performance under poor conditions may not indicate better welfare, but rather a shift in motivation for food rewards.

## Introduction

Farm animal welfare depends largely on how animals interact with and adapt to their environments. When animals are unable to predict or control environmental events, sustained mismatches between expected and actual sensory inputs can lead to stress and negative affective states (Colditz 2018). In laying hens, barren and restrictive environments reduce opportunities for control and agency, thereby affecting their welfare (Baxter 1994; Duncan 2001).

Barren environments can also affect cognition (Tahamtani *et al.* 2015; Ferreira *et al.* 2021; Dumontier *et al.* 2023). For instance, hens reared in cages show slower learning, reduced working memory (Tahamtani *et al.* 2015), and lower spatial performance compared to aviary-reared hens (Dumontier *et al.* 2023), highlighting the sustained effects that lack of environmental complexity can trigger.

Understanding the cognitive abilities of chickens is essential for improving welfare in production systems, as it provides insights into how animals perceive and respond to their environment (Ferreira *et al.* 2021). How animals anticipate and interpret events can influence their welfare, and impairments in these processes may lead to welfare issues (such as profound cognitive deficits) that would otherwise remain unnoticed (Lecorps & Weary 2024). Cognitive research can also identify mismatches between rearing conditions and animals’ adaptive capacities and use shifts in performance as indicators of changes in motivation (Nawroth *et al.* 2019).

Individual behavioural differences that are consistent over time, known as animal personality (Gosling 2008), can also influence how animals process and respond to their environment, including in cognitive tasks. Personality is often described along a proactive-reactive continuum: proactive individuals show active coping and low HPA reactivity, whereas reactive ones display passive behaviour and higher corticosterone levels (Cockrem 2007). In poultry, more fearful and reactive birds have shown better cognitive performance than proactive ones, suggesting personality-linked cognitive styles (de Haas *et al.* 2017a; Ferreira *et al.* 2019). This effect, however, appears context-dependent, as the influence of housing on behaviour and affective state can vary with individual traits (Bari *et al.* 2021; Campbell *et al.* 2021; Ulans *et al.* 2024).

Despite growing evidence that housing conditions shape cognition and emotions in laying hens, little is known about how transitions to barren environments affect cognition later in life. Pullets may be reared in aviary systems but later transferred to restrictive cages (Du *et al.* 2022), a transition associated with frustration, feather pecking, and poor adaptation (Janczak & Riber 2015). The aim of this study was to evaluate whether a transition to a battery cage affects spatial memory performance in laying hens, and whether individual personality traits modulate this effect. We also assessed persistence in search behaviour after reward depletion to explore potential differences in behavioural flexibility/extinction-related processes. We hypothesised that hens moved to battery cages would show poorer performance in the spatial memory task compared to those in a cage-free environment. Furthermore, we expected fearful birds to be more negatively affected by restrictive housing.

## Materials and methods

### Ethical considerations

The procedures described were reviewed and approved by the Universidad Austral de Chile Institutional Animal Care and Use Committee (488/2023). Birds were sourced from a commercial flock destined for a cage-free housing system (flock size: 13,500 birds). As part of the experimental design, half of the hens were transferred to battery cages for 82 days to investigate the effects of reduced environmental complexity, which meant reproducing commercial battery housing conditions without additional enrichment. Although refinement options were limited by the need to emulate these conditions, all behavioural protocols were designed to minimise stress and avoid unnecessary handling. A standardised welfare assessment was conducted daily, including checks for behavioural abnormalities (e.g. reduced activity, panting, persistent vocalisation), physical indicators (e.g. plumage damage, wounds, lameness), and a predefined experimental endpoint used to determine whether a bird required intervention or exclusion from the study. At the end of the study, all birds were rehomed to private households, where they continue to be cared for as backyard companion chickens. This study followed the ARRIVE guidelines for reporting animal research (Percie du Sert *et al.* 2020).

### Study animals and initial management

The study involved 60 Lohman Brown laying hens reared from hatch to 16 weeks of age in a commercial cage-free aviary. Beak trimming was performed at the farm of origin as part of routine husbandry (infrared beak trimming at 1 day of age). At 16 weeks of age, prior to the onset of lay, the birds were transported to the experimental poultry unit at the Universidad Austral de Chile.

The sample size was constrained by facility capacity and consistent with previous studies achieving sufficient statistical power for comparable effect sizes (Tahamtani *et al.* 2015; Campbell *et al.* 2019; Anderson *et al.* 2021).

### Experimental timeline and housing conditions

On arrival (16 weeks old; day 1 of the study), the hens were placed in two identical aviary pens (4 × 2 × 2 m; length × width × height), each accommodating 30 birds (space allowance: 2,667 cm² per hen). The pens had wooden floors, wire-mesh walls, and a two-tier layout connected by wooden ramps set at a 40° incline. The lower level contained a wood-shavings foraging and dust-bathing area (4 × 0.8 m; length × width), three yellow bowl drinkers, and a circular feeder (27 cm diameter; 8 kg capacity). The upper tier housed three individual nest boxes (47 × 29 × 47 cm), an additional foraging area with wood-shavings and sand, and a wooden perch unit with three horizontal bars at different heights.

From day 1 to day 29 (ages 16–18 weeks), all hens remained under cage-free conditions in the aviary pens for acclimation. During this pre-treatment phase, personality testing was conducted. Feed and water were supplied *ad libitum* using the same commercial layer diet provided throughout the experiment.

On day 29 (age 18 weeks), the flock was randomly split into two housing treatments:

1. Cage-free pens: 15 hens remained in each aviary pen, doubling the available space per bird compared with the acclimation phase.
2. Conventional battery cages: the remaining 15 hens from each pen were moved into eight cages (48 × 38 × 38 cm), with four hens per cage (450 cm² per bird, matching standard commercial density; Shields & Duncan 2009).

The cages were arranged in two rows of four, fitted with nipple drinkers along a central water line and a horizontal feeder (49 × 10 cm). Floors were sloped wire-mesh to allow egg collection. All housing (pens and cages) was located in the same room, separated only by a curtain (see Supplementary material; https://doi.org/10.5281/zenodo.15880771), allowing birds auditory and olfactory contact across treatments. The assigned housing was maintained for 84 days, until day 111 of the experiment.

### Study procedure

**Figure 1.**
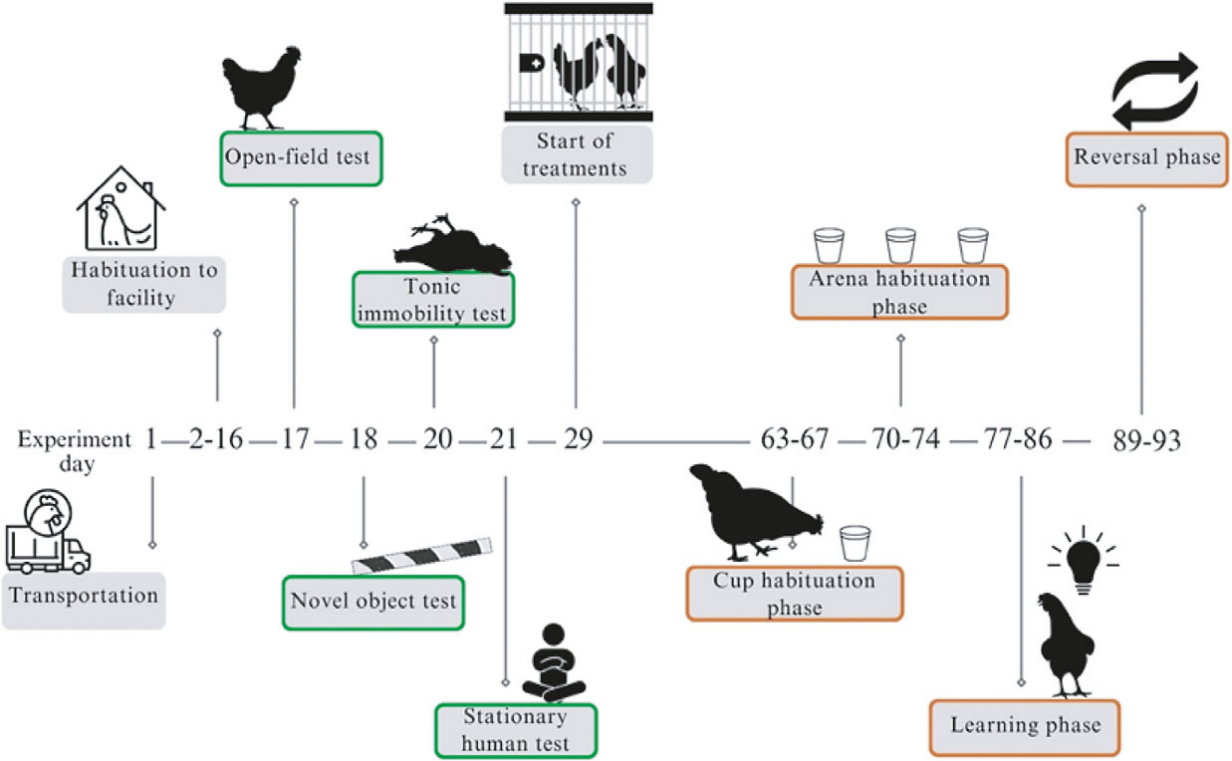
Experimental timeline. Chickens (n = 60) arrived at the research facility when 16 weeks old (day 1 of the experiment), and were then habituated to the facility, before being subjected to behavioural tests (days 17 to 29), and placed in two different treatments starting on day 29 (aviary vs barren cages). Birds were then individually tested for spatial cognition.

The timeline of the experiments is shown in Figure 1. For all tests, hens were recorded using DH-HAC-HDW1200EM-A cameras (Zhejiang Dahua Technology, Hangzhou, China).

#### Personality tests

Four personality tests were carried out on different days, to assess individual differences in fearfulness, exploratory behaviour, and social avoidance.

The Open Field Test (day 18; adapted from de Haas *et al.* 2017a,b) was conducted in a 1 × 1 × 1 m wooden arena with three solid walls and a wire-mesh front and top. The room remained dark during placement, and lights were turned on once the bird was inside and the experimenter exited. For 3 min, we recorded latency to walk (time until two consecutive steps), number of vocalisations, and quadrants crossed, providing a measure of general fearfulness and exploratory tendency.

The Novel Object Test (day 19; adapted from Brantsæter *et al.* 2017; Larsen *et al.* 2018) was conducted in the same arena. Each hen was exposed for 2 min (Brantsæter *et al.* 2017) to a 50-cm stick with multi-coloured ribbons, and latency to walk, number of vocalisations, quadrants crossed, and latency to approach the object were recorded. These measures indicate neophobia and exploratory motivation.

The Tonic Immobility Test (day 21; adapted from de Haas *et al.* 2017a) was performed in a separate room. Hens were gently placed supine in a cradle, and immobility was induced by light pressure on the sternum and legs while covering the head. Hens that remained immobile for more than 10 s were considered as successfully induced, and their latency to stand up was recorded (maximum 300 s) as an indicator of passive fearfulness. A maximum of three inductions were performed.

The Stationary Human Test (day 22; Brantsæter *et al.* 2017) was conducted in a familiar 3.4 × 1.9 m room and lasted 3 min. Each hen was placed individually in the room and left alone for 30 s. Then, an unfamiliar human female entered, paused briefly at the door, and sat quietly within a marked 1-m semi-circle. We recorded birds’ latency to approach, the time spent within 1 m of the person, and the number of vocalisations, as measures of fear of humans or social avoidance.

#### Spatial memory test

From day 63 to day 95, birds were tested in a modified hole-board test based on Dumontier *et al.* (2023). The test included four phases: Cup Habituation Phase; Arena Habituation Phase; Learning Phase; and Reversal Phase (Figure 1). For clarity, test progression is shown using consecutive test days (Day 1–15) in figures and results.

Of the 60 birds initially enrolled, four hens were removed due to sickness (three from battery and one cage free). Additionally, birds were excluded if they were subjected to more than two human errors in the configuration of the test or if they failed to eat any sweetcorn during the habituation phase. Consequently, 51 hens (25 housed in battery cages and 26 housed in cage-free pens) were retained for the final analysis.

**Figure 2.**
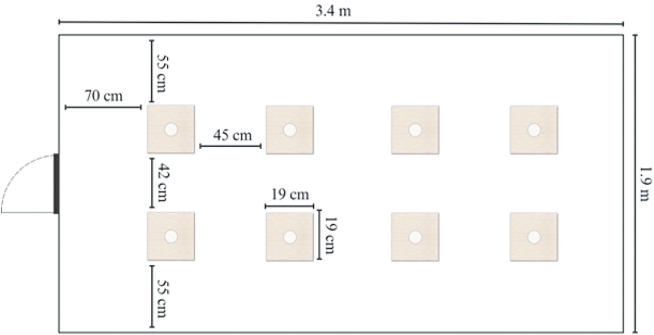
Schematic representation of the hole-board arena (top view) used for testing hens (n = 60). The diagram shows the layout of the hole-board arena, including the positions of the cups and their corresponding plywood squares, arranged in a 2 × 4 matrix within the 3.4 × 1.9 m space.

The test was conducted in a 3.4 × 1.9 m room with a grey-painted concrete floor. Eight plywood squares (19 × 19 cm) were arranged in a 2 × 4 matrix (Figure 2), each with a cup glued to the centre of the plywood. The white cups, measuring 2.5 cm in depth and 4.5 cm in diameter, were used to hold the sweetcorn treats during the test. A ceiling-mounted camera was used to record the trials.

*1. Cup Habituation Phase.* During this phase, hens were introduced to the cups used in the arena. For five days, cups containing sweetcorn were placed in the hens’ home environment. For cage-free hens, the cups were positioned in the home pen, while for battery-caged hens, the cups were placed in the feeder area.

*2. Arena Habituation Phase.* Over the next five days, hens were individually exposed to the arena for 5-min sessions. During these sessions, all eight cups were baited with a single sweetcorn kernel to encourage exploration. Each hen was placed at the arena entrance, the door was closed, and the hen was allowed to explore freely for the duration of the session. Hens that failed to find and eat all sweetcorn within the five-day habituation period were excluded from the test.

*3. Learning Phase.* The learning phase lasted ten days, with hens placed in the arena for 5 min once per day. During this phase, only three of the eight cups were baited with one sweetcorn each. The baited cups remained consistent throughout the phase. Four different configurations of baited cups were used, and each hen was randomly assigned one configuration. The assignments were balanced across treatments.

*4. Reversal Phase.* The final phase lasted five days. During this phase, the configuration of the baited cups was changed from the learning phase, and the new configuration remained consistent for all five days. This phase tested the hens’ ability to learn the new reward locations.

At the start of each testing session, the hen was placed at the arena entrance, and the door was closed. Hens were given 5 min to find the baited cups. Once all three sweetcorn were consumed, hens remained in the arena until the 5-min session ended. This post-consumption interval was considered the post-reward period and hens’ behaviours including comfort-like behaviours such as preening, wing flapping and explorative behaviours (Weeks & Nicol 2006; Abeyesinghe *et al.* 2021) were recorded (Table 1).

**Table 1.** Behavioural variables registered during the spatial memory task. Measures were collected across the learning, reversal, and post-reward phases as indicated

Variable	Description	Phase
Latency to all rewards	Time spent before completing finding the three rewards (s)	Learning and reversal
Total cups visited	All baited and unbaited cup visits (visits per session)	Learning and reversal
Unique cups visited	Distinct cups explored within session (cups per session)	Learning, reversal, post-reward
Revisits to baited cups	Repeated visits to baited cups already emptied (visit per session)	Learning, reversal, post-reward
Preening	Self-grooming behaviours (events per s)	Post-reward
Flight behaviour	Wing-flapping or short escape-like jumps (events per s)	Post-reward
Foraging behaviour	Ground scratching using both legs (events per s)	Post-reward

From hens’ behaviours, following the approach described by Dumontier *et al.* (2023), we calculated three cognitive indices commonly used in hole-board tasks. These scores were calculated based on the hens’ choices until all baited cups had been found (excluding the post-reward period). Higher values in these metrics indicate better spatial memory performance and more effective foraging strategies.

- *Working Memory (WM)* reflects short-term memory by measuring the ability to avoid revisiting baited cups within a trial. It is calculated as the proportion of successful visits (to baited cups) relative to the total visits to those cups.
- *General Working Memory (GWM)* also reflects short-term memory and indicates the capacity to avoid revisiting cups that have already been visited. It is calculated as the ratio of unique cups visited to the total number of visits within each session.
- *Reference Memory (RM)* captures long-term memory, representing the hen’s ability to remember where baited cups are located between trials. It is calculated by dividing the number of visits to baited cups by the total visits across all cups.

### Statistical analysis

The study used a two-treatment, between-subject design comparing cage-free and battery-cage housing systems. All analyses were performed in R 4.2.2 using the individual hen as the unit. Video scoring was performed by an observer blind to the treatment allocation using BORIS (Friard & Gamba 2016). The complete dataset is available in Zenodo at https://doi.org/10.5281/zenodo.17306200.

The individual hen was the statistical unit (final; n = 51; 25 battery, 26 cage-free). From the initial 60 hens, ten were excluded: one due to a beak deformity that impaired sweetcorn intake, three for not meeting the habituation criterion, two due to illness during testing, and four for repeated human technical errors in bait placement.

#### Personality traits

To obtain more reliable estimates of personality traits, behaviours reflecting the same underlying motivation and measured across different tests were aggregated, as recommended in previous research (Fleeson 2001; Lecorps *et al.* 2018). Such aggregation reduces context-specific noise and increases the ability to detect consistent individual differences, providing a better assessment of personality than single measures from one context (Lecorps *et al.* 2018). For example, latencies to walk in the OFT, NOT, and HT were averaged into a single value per hen. In total, three aggregated variables were used: latency to walk; number of vocalisations; and quadrants crossed.

Before analysis, all variables were standardised by dividing by test duration (0–1 range) and then scaled. PCA was run using the *psych* package (Revelle 2025) and visualised with *factoextra* (Kassambara & Mundt 2020). Components were retained based on Eigenvalues >1 and the scree plot, and varimax rotation was applied for clarity. Variables with loadings ≥ |0.5| defined each component, which were labelled according to their main behavioural traits.

Although personality tests were performed before treatments were applied, to confirm that personality traits were balanced across treatments, linear models were run for each principal component using the lm() function.

#### Spatial cognition performance

For each dependent variable — latency to find the first corn, latency to find all corns, Working Memory Ratio (WM), General Working Memory Ratio (GWM), and Reference Memory Ratio (RM) — linear mixed-effects models (LMMs) were fitted using restricted maximum likelihood estimation with the nlme package (Pinheiro *et al.* 2021) in R version 4.2.2.

Two sets of models were constructed. The first included treatment, day, and their interaction as fixed effects. The second included treatment, personality (PC1–PC3), and their interaction. Hen was included as a random effect in all models to account for repeated measures. Non-significant interactions were removed, and models were rerun accordingly. Each test phase (learning day 77 to 86 and reversal day 89 to 93) was analysed separately. This two-step approach was chosen to maintain model parsimony and to evaluate the effect of personality on treatment responses without overparameterising the models.

To evaluate the effects of the change in bait configuration, we analysed the phase change from the last day of the learning phase (day 13) to the first day of the reversal phase (day 14). For each treatment, paired *t*-tests were conducted to compare performance across these two days.

Model assumptions (normality, homoscedasticity) were verified through visual inspection of residuals. When assumptions were violated, a logarithmic transformation or square-root transformation was applied.

The behaviours recorded during the post-reward period (defined as the time after the hen had consumed all three baited corns) were standardised by dividing their frequency by the total post-reward time for that hen on each trial. These standardised frequencies were then analysed using the same linear mixed-effects model (LMM) described above, with treatment, day, and their interaction as fixed effects, and hen ID as a random effect to account for repeated measures. The same for models including personality traits. Each variable was analysed separately.

## Results

### Personality

The PCA explained 78% of the variance across three dimensions. The first (34.3%) reflected proactive-exploratory tendencies, characterised by greater locomotion, vocal activity, and shorter latencies to initiate movement. The second (26.9%) captured fearfulness, with delayed approach to novelty and reduced proximity to humans. The third (16.9%) reflected immobility, with longer tonic immobility durations indicating reliance on passive defence. Personality scores did not differ between treatment groups (*P* > 0.05).

### Spatial memory test

#### Learning phase

Latency to find all corns decreased significantly over days (*F*₁,₄₄₇ = 15.57; *P* < 0.001), indicating that hens became progressively faster at locating all baited cups as the testing period progressed. Housing treatment had no effect, and no treatment × day interaction was detected. More fearful hens (PC2) took longer to find all baits (*F*₁,₄₀ = 4.99; *P* = 0.03), whereas there was a trend for more exploratory hens (PC1) to complete the task more quickly (*F*₁,₄₀ = 3.35; *P* = 0.07). No significant effects were found for PC3 or any interaction terms.

**Figure 3.**
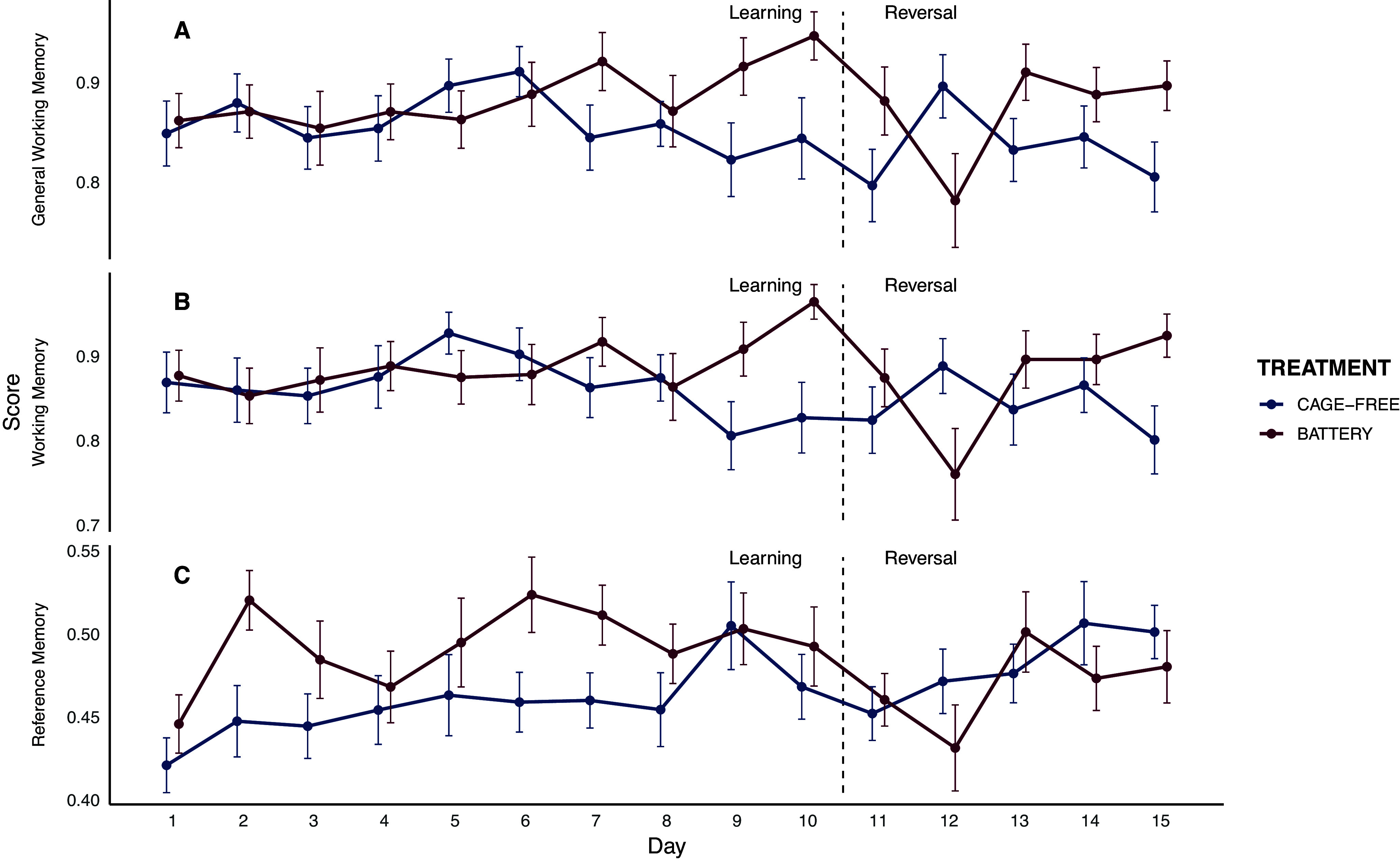
General working memory (A), reference memory (B), and working memory (C) scores across test days for hens housed in battery cages (red; n = 25) and cage-free systems (blue; n = 26). The graph displays both the learning phase and the reversal phase, separated by a dashed line. Error bars represent the standard error of the mean (SEM).

Battery hens reduced revisits to baited cups more rapidly than cage-free hens across days, indicating faster improvement in working memory (treatment × day: *F*₁,₄₁₅ = 6.37; *P* = 0.01). They also avoided revisiting previously explored cups more efficiently over time, reflecting higher general working memory (treatment × day: *F*₁,₄₁₆ = 5.87; *P* = 0.01). More fearful hens (PC2) revisited baited and previously visited cups more often, resulting in lower working memory (*F*₁,₃₇ = 3.64; *P* = 0.06) and general working memory scores (*F*₁,₃₆ = 4.49; *P* = 0.04). No effects were found for other personality traits or their interactions with housing. For long-term spatial memory, battery hens visited baited cups more selectively than cage-free hens overall (reference memory; *F*₁,₄₀₈ = 3.88; *P* = 0.04), and performance improved across days in both treatments (*F*₁,₄₀₈ = 4.49; *P* = 0.03; Figure 3). No effects were found on treatment × day interaction or personality.

#### Reversal phase

For working memory ratio, cage-free hens maintained stable performance (*P* = 0.96), whereas battery-caged hens showed a significant decline (*t*[24] = 2.23; *P* = 0.036). General working memory and reference memory scores did not change (all *P*s > 0.05; Figure 3).

Battery hens were slower to find all baited cups compared to cage-free hens at reversal (*F*₁,₁₉₅ = 3.95; *P* = 0.04). There was also a trend toward a treatment × day interaction (*F*₁,₁₉₇ = 2.93; *P* = 0.08), suggesting that although battery hens initially performed more slowly, their rate of improvement over time may have been greater.

When analysing performance across the entire reversal phase, different trends emerged. For WM, there was a significant treatment × day interaction (*F*₁,₁₉₆ = 3.99; *P* = 0.040), reflecting a progressive improvement in battery hens, whereas no main effects of treatment, day, or personality traits were found. GWM showed no significant effects of treatment, day, personality traits, or their interactions. In contrast, RM increased significantly over days (*F*₁,₁₉₇ = 6.14; *P* = 0.010), independently of treatment or personality effects (Figure 3).

#### Post-reward period

During the learning phase, both the number of visits to previously baited cups (*F*₁,₄₄₀ = 49.73; *P* < 0.001) and the total number of cups visited (*F*₁,₄₄₀ = 49.74; *P* < 0.001) increased significantly over time, with no effects of treatment or personality traits. In the reversal phase, battery hens showed a significant increase in visits to previously baited cups (*F*₁,₁₉₂ = 13.84; *P* < 0.001). A comparable pattern was observed for total visits (*F*₁,₁₉₂ = 13.83; *P* = 0.0003), with both measures rising over time in caged hens. No significant effects of personality traits were detected for either measure.

**Figure 4.**
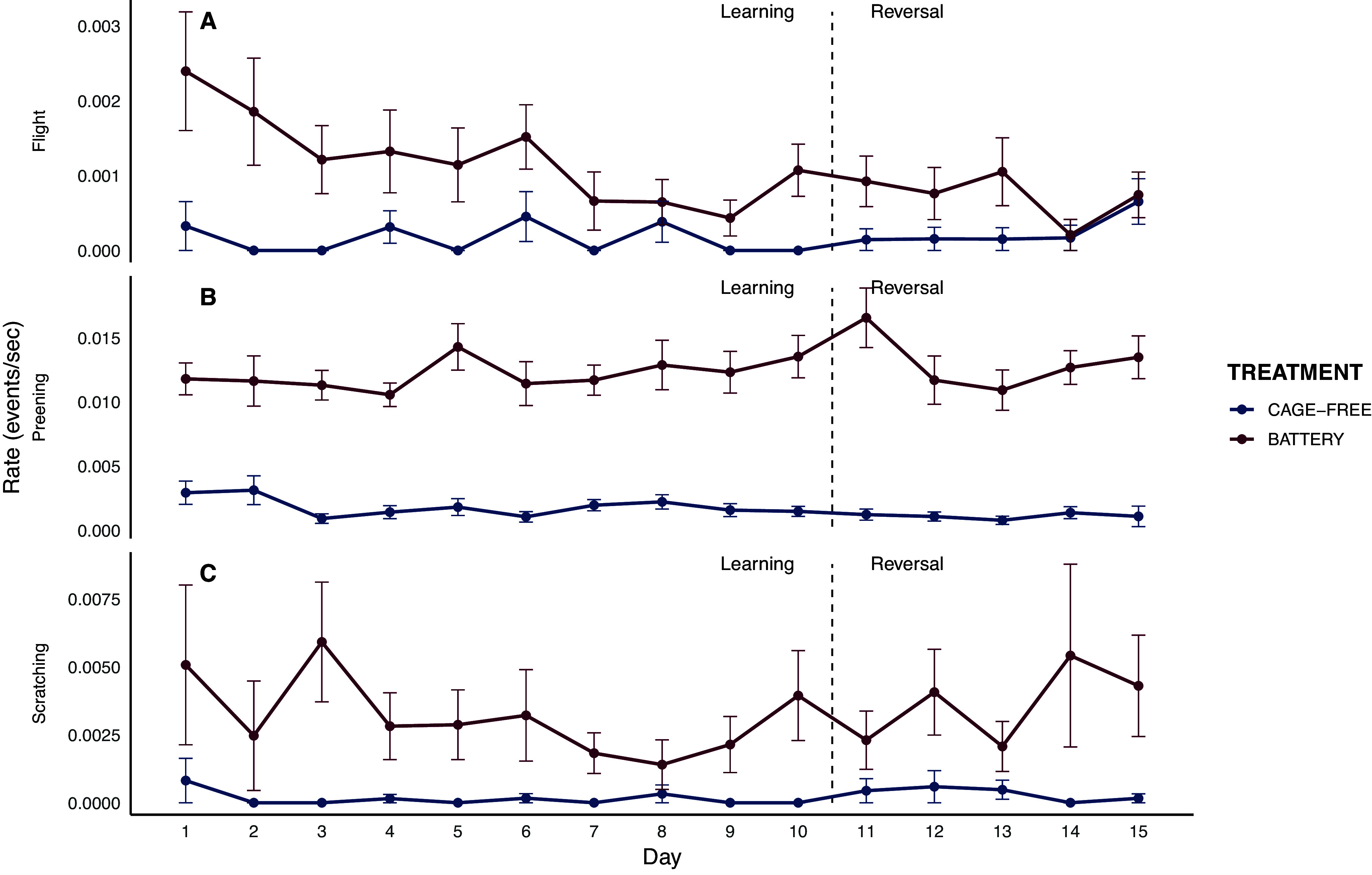
Rates of flight, preening, and scratching behaviours during the post-reward period across trial days for hens housed in battery cages (red; n = 25) and cage-free systems (blue; n = 26). Data are shown separately for the learning and reversal phases, divided by a vertical dashed line. Error bars indicate the standard error of the mean (SEM).

During the learning phase, battery hens performed significantly more flight behaviour than cage-free hens (*F*₁,₂₇₅ = 23.58; *P* < 0.001). Importantly, there was a significant treatment × day interaction (*F*₁,₄₁₃ = 6.61; *P* = 0.010), indicating that the initial difference between treatments decreased over sessions. In the reversal phase, battery hens also showed more flight events (*F*₁_,191_ = 4.44; *P* = 0.03). Preening was consistently higher in battery hens across both phases (learning: *F*_1,231_ = 48.89; *P* < 0.0001; reversal: *F*_1,191_ = 9.18; *P* = 0.002). Scratching behaviour was also more frequent in battery hens than in cage-free hens during learning phase (*F*_1,152_ = 9.39; *P* = 0.002). Personality had no effect on any of these behaviours (Figure 4).

## Discussion

Overall, our results showed that battery hens performed better in the spatial memory task. They were also more active during the post-reward period, making more visits to empty cups and showing more foraging and comfort-like behaviours than cage-free hens. More fearful hens, regardless of housing, showed poorer short-term memory performance, as reflected by a higher tendency to revisit previously baited or already visited cups, whereas more exploratory individuals found the baited cups more quickly. Together, these findings indicate that both housing conditions and individual personality traits can influence learning and memory performance in laying hens.

Our hypothesis that battery hens would be outperformed by cage-free hens in the spatial memory task due to housing-induced cognitive deficits was not supported. Instead, battery hens improved faster than cage-free hens over time in WM and GWM, and they consistently showed better reference memory. These results contrast with studies showing cognitive benefits of enriched environments in poultry and other species (e.g. Salvanes *et al.* 2013; Campbell *et al.* 2018; Dumontier *et al.* 2023). Although RM values were modest perhaps due to the short acquisition phase, hens did show significant improvement across days. Importantly, housing-related differences in RM appeared early and remained consistent, indicating robust group-level effects regardless of the absolute level of learning.

It is possible that, for battery hens, the spatial memory task itself acted as a form of cognitive enrichment, providing stimulation otherwise absent from their daily environment (Meehan & Mench 2007). At the same time, chronic housing restriction may have increased the motivational salience of food rewards, thereby enhancing engagement with the task. Evidence from poultry and other species supports this idea: hens show anticipatory arousal when rewards are visible but inaccessible (Davies *et al.* 2014), acutely stressed hens approach ambiguous cues more quickly in judgment bias tests (Hernández *et al.* 2015), stress can enhance reward-based learning in rodents (Schettino *et al.* 2024), and calves housed in barren environments approach milk rewards faster than those reared in enriched settings (Suchon *et al.* 2025).

Together, these findings suggest that barren housing can heighten reward motivation, which (when combined with the cognitive stimulation provided by the task) may explain the superior memory performance of battery hens. Heightened incentive salience is known to strengthen attentional allocation and cue-driven control of behaviour (Berridge & Robinson 1998), and welfare frameworks propose that behavioural efficiency and persistence reflect the functional state of reward-evaluating mechanisms under challenge (Spruijt *et al.* 2001). This mechanism provides a coherent explanation for the superior working memory, general working memory, and reference memory performance observed in battery hens.

Bayesian models of conditioning predict that learning accelerates when outcomes deviate from prior expectations, as ‘surprise’ signals a change in contingencies and prompts animals to update predictions (Courville *et al.* 2006; Lecorps & Weary 2024). For battery hens, the spatial memory task may have been both novel and rewarding, combining food motivation with the positive affective impact of unexpected stimulation, thus fostering engagement and learning. In contrast, cage-free hens, accustomed to richer environments and greater space, may have experienced the test more negatively, as isolation and spatial restriction could have elicited frustration or stress. For these birds, food rewards were likely less salient, reducing motivation and task engagement. This interpretation aligns with their lower performance across memory metrics and the absence of change between learning and reversal phases. A similar pattern has been reported in rodents, where enriched animals attribute less incentive salience to food-associated cues than those from barren conditions (Beckmann & Bardo 2012).

The post-reward period, during which hens remained in the arena after consuming all rewards, provided an opportunity to assess behavioural responses to the absence of expected reinforcement. During this phase, battery hens showed higher levels of preening, wing-flapping, and scratching. These behaviours may reflect the expression of activities normally constrained in cages, but they have also been described as displacement or arousal-related responses in birds. For example, increased preening has been associated with elevated feather corticosterone levels in open-field tests (Kozak *et al.* 2019). Importantly, however, such behaviours are not explicit indicators of negative affect and may also reflect engagement with novelty or enjoyment of increased space relative to home-pen restrictions.

Battery hens also made more visits to cups that had previously been baited and showed higher exploration compared to cage-free hens. This was particularly evident in the reversal phase, where caged hens became increasingly persistent in visiting unrewarded cups. Such persistence suggests a deficit in extinction learning possibly driven by a heightened reward-related motivation. Stress has been shown to impair extinction learning, maintaining previously reinforced behaviours (Rodberg 2020). In our study, the restrictive conditions experienced by battery hens may have similarly enhanced reward motivation and reduced cognitive flexibility, leading to persistent search behaviour during the post-reward period.

Most hole-board studies have focused on how early-life housing affects cognition. For instance, Dumontier *et al.* (2023) and Tahamtani *et al.* (2015) reported that hens reared in enriched environments outperformed cage-reared birds, even when later tested under similar conditions. By contrast, our study manipulated housing in adulthood, after all hens had been reared cage-free. Early enrichment is more likely to produce lasting neural and behavioural changes, whereas enrichment during adulthood may yield more transient, functional effects (Zentall 2021). Patzke *et al.* (2009) found only mild hippocampal effects of housing changes imposed in adulthood, suggesting limited neural sensitivity to environmental downgrading at this stage. This aligns with our finding that battery-housed hens did not show the cognitive impairments typically associated with restrictive rearing conditions. In our case, prior exposure to a cage-free environment may have buffered battery hens from the expected cognitive costs of restriction. Rather than showing impairment, these birds appeared more motivated by food rewards. Future studies could employ post-reward-based tasks to better isolate cognitive effects from motivational ones.

In addition to housing effects, our findings highlight the role of individual differences, with both fearfulness and exploration traits influencing cognitive performance. In birds, contrasting results have been reported: less explorative junglefowl performed better in a reversal task (Zidar *et al.* 2018), while more fearful chickens showed better spatial performance in a within-day test (Ferreira *et al.* 2019). Nordquist *et al.* (2011) found no clear differences. In our study, more fearful hens had worse WM and GWM, and took longer in finding all the baited cups. These effects were independent of housing. Our results are in accordance with those of de Haas *et al.* (2017b), who found that fearful chickens learned more slowly, relied on rigid strategies, and reacted more strongly to task changes or unrewarded cues, patterns that may explain the learning difficulties experienced by our fearful hens. However, contrary to our expectations, personality did not interact with housing. This differs from studies in pigs, where coping style and housing interacted to affect behaviour (Bolhuis *et al.* 2004). The lack of interaction here may reflect that the housing change had limited impact, or that altering housing earlier in life would have affected vulnerable individuals more strongly.

Personality is defined as behavioural consistency across time and contexts (Réale *et al.* 2007). A limitation of the present study is that personality was assessed at a single time-point, which restricts inference about temporal stability. Although our single time-point assessments limit inference about temporal stability, the assays used here are widely validated in poultry as indicators of stable personality dimensions (Forkman *et al.* 2007). Measuring behaviour across multiple contexts provides cross-situational information about individual differences, supporting construct validity when repeated measures are not feasible (Réale *et al.* 2007; Carter *et al.* 2013). Accordingly, single time-point multi-test batteries are commonly used to characterise personality in laying hens (de Haas *et al.* 2017a; Zidar *et al.* 2017).

Some features of the task design may have influenced how hens engaged with the spatial memory test. First, the baited cups may have been partially visible, allowing hens to potentially rely on visual rather than spatial cues, which could explain the consistently high initial WM scores (~0.87). Second, remaining in the arena after consuming all rewards may have induced mild frustration or disengagement, reducing motivation in subsequent trials. These factors do not invalidate the task but highlight how subtle design elements can influence cognitive performance.

### Animal welfare implications

Most laying hens worldwide are kept in barren, restrictive housing, making research on these systems ethically and scientifically relevant despite legislative progress in some parts of the world. Understanding how such environments affect cognition and affective states remains crucial for improving global welfare standards. This study shows that transitioning to a restrictive housing can alter motivation for rewards likely to explain both the heightened cognitive performance and the deficit in extinction learning. Recognising these cognitive and emotional costs is essential to interpret behaviour correctly and to design housing systems that promote not only productivity but also mental well-being, supporting evidence-based policies for the development of housing systems that better meet behavioural and emotional needs.

## Conclusion

In summary, our findings highlight the complex interplay between housing conditions, individual personality traits, and cognitive performance in laying hens. Contrary to our expectations, hens housed in barren battery cages outperformed their cage-free counterparts in a spatial memory task. Importantly, this pattern should not be interpreted as evidence of superior welfare in caged birds. Rather, the observed performance differences are most plausibly explained by heightened reward motivation and increased attentional engagement with the task, rather than by enhanced cognitive abilities. In addition, battery hens showed greater persistence in visiting previously rewarded cups during the non-reward period, suggesting altered extinction learning driven by elevated incentive salience.

Personality traits (particularly fearfulness) consistently influenced performance, independently of housing. These findings highlight the importance of considering both environmental and individual-level variables when evaluating animal cognition and welfare. They also caution against interpreting cognitive performance as a straightforward proxy for positive animal welfare, especially when motivational and affective states may be confounded with task engagement.

## Supporting information

10.1017/awf.2026.10084.sm001 Calderón-Amor et al. supplementary material Calderón-Amor et al. supplementary material

## Supplementary material

The supplementary material for this article can be found at http://doi.org/10.1017/awf.2026.10084.
