# Supplementary figures and images for: Moving hens into cages affects cognitive performance, extinction learning and motivation for rewards
Source: Anim Welf. 2026 Apr 14;35:e29. doi: 10.1017/awf.2026.10084 (PMC13107361; doi:10.1017/awf.2026.10084)

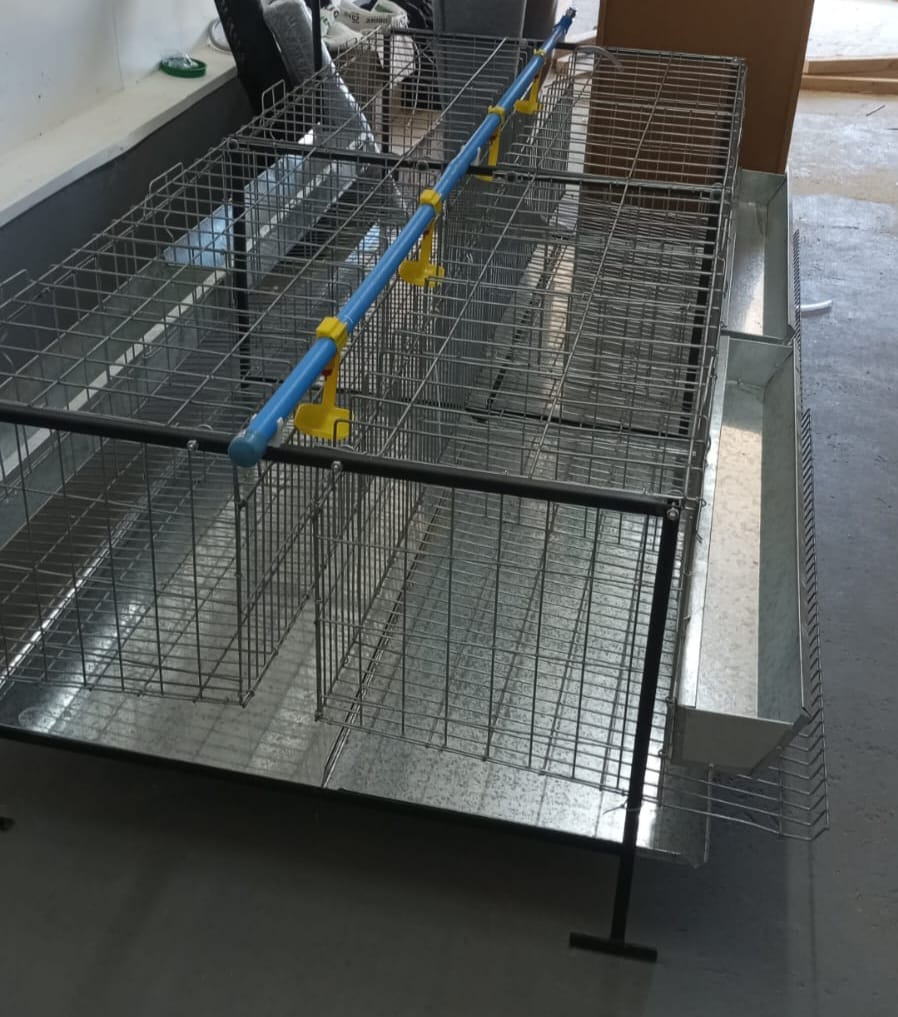

Supplement: Calderón-Amor et al. supplementary material [file S0962728626100840sup001.zip › Battery cages.jpg]
